# Radiographic Follow-Up during Orthodontic Treatment for Early Diagnosis of Sequential Supernumerary Teeth

**DOI:** 10.1155/2016/3067106

**Published:** 2016-05-24

**Authors:** Selma Sano Suga, Paula de Castro Kruly, Talissa Mayer Garrido, Marise Sano Suga Matumoto, Uhana Seifert Guimarães Suga, Raquel Sano Suga Terada

**Affiliations:** State University of Maringá, Avenida Mandacaru, 1550 Bairro Mandacaru, 87080-000 Maringá, PR, Brazil

## Abstract

Most supernumerary teeth are impacted and asymptomatic.* Objective.* The aim of this paper is to describe two cases of sequential development of supernumerary teeth in the mandibular premolar region, identified during orthodontic treatment.* Reports.* The first case describes the radiographic follow-up of a female patient that presented a supernumerary tooth at the age of 9 years and 10 months in the right mandibular premolar region, followed by a further supernumerary tooth in the left mandibular premolar region identified at the age of 11 years and 3 months. In the second case, the radiographic follow-up of a male patient demonstrated 3 supernumerary teeth in the premolar region at the age of 16 years. During orthognathic surgery planning at the age of 20 years and 5 months, a supplemental supernumerary tooth was found in the left mandibular region.* Conclusion.* Considering the late developing of supernumerary premolars, appropriate follow-up with panoramic radiographs of patients with previous experience of supernumerary teeth is essential for early diagnosis of supplemental premolars to prevent possible complications.

## 1. Introduction

Supernumerary teeth can affect the development of occlusion [1, 2], cause root resorption of adjacent teeth, and induce pathological changes such as cysts [3]. The prevalence of third premolars is relatively low (0.29% of the population), representing between 8 and 9.1% of all supernumerary teeth [4]. Approximately 75% of all third premolars remain impacted [5]. As they tend to remain trapped and are almost always asymptomatic, radiographic diagnosis is usually incidental [6]. Therefore, early diagnosis performed with routine radiographic examinations before, during, and after clinical or orthodontic treatment is essential to identify these alterations, which can negatively affect the normal development of occlusion [2, 7].

There are few reports in the literature on sequential supernumerary teeth in the premolar region [8]. Because they tend to occur after the normal tooth development period, they can interfere with orthodontic treatment.

Although not clearly defined in the literature, supernumerary teeth have been reported as being “nonsequential,” when diagnosed on a single occasion, and as “sequential,” when they are found at different times over a period of an individual's life [9, 10]. The incidence of supernumerary teeth in the permanent dentition has been reported to range between less than 1% and 4% [9] and 1% and 3.5% [3], while in the deciduous dentition it ranges between 0.3% and 0.8% [9] and 0.3% and 0.6% [3]. Supernumerary teeth are more frequently reported in males than in females in a proportion of 2 : 1 [7]. In previous reviews of the literature on supernumerary teeth, the mandibular premolar region was found to be the most common site of occurrence [5, 11].

Therefore, the aim of this paper is to report on two cases of sequential development of supernumerary teeth in the mandibular premolar region and discuss the importance of the clinical and radiographic follow-up during orthodontic treatment.

## 2. Case Reports

**Figure 1:**
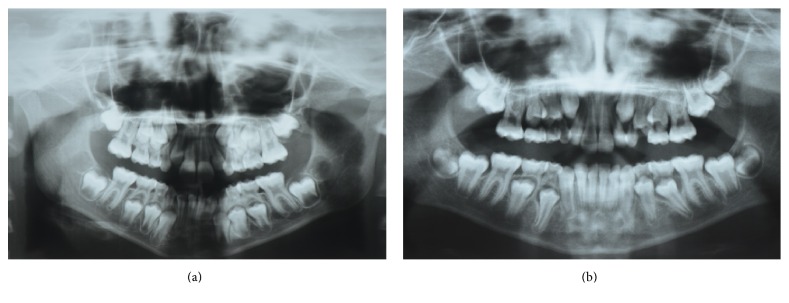
(a) Panoramic radiograph of the patient aged 7 years and 3 months and (b) 9 years and 4 months with no evidence of supernumerary teeth.

*Case  1.* A female, leucoderma, Brazilian patient, aged 7 years and 3 months, presenting mouth breathing and skeletal Class II, division 2 malocclusion, sought a private clinic for orthodontic treatment. The initial panoramic radiograph revealed no abnormalities in the dentition (Figure 1).

**Figure 2:**
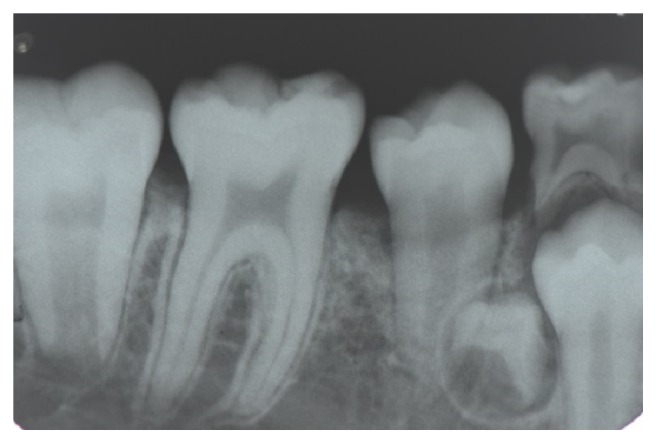
Periapical radiograph of the patient aged 9 years and 10 months showing early formation of a supernumerary tooth in the right mandibular premolar region.

**Figure 3:**
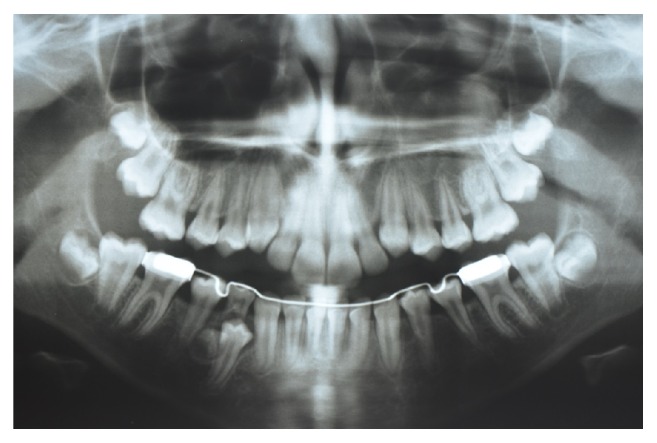
Panoramic radiograph of the patient aged 10 years 5 months showing early formation of a supernumerary tooth in the right mandibular premolar region.

Orthodontic treatment started two years later, when the patient was aged 9 years and 4 months. The corrective phase of the treatment occurred after the interceptive phase. For the treatment of the Class II malocclusion, an orthopedic expansion device across the upper and lower arches was placed over a period of 12 months. After this period, a lingual bar was adapted, and a follow-up radiograph was required because tooth 84 exhibited no physiological mobility and was the last deciduous teeth to remain in the oral cavity. Control radiographs obtained at 31 and 38 months after the first initial radiographic assessment revealed the early formation of a supernumerary tooth in the right mandibular premolar region (Figures 2 and 3). There were no reports of supernumerary teeth in the family. Due to its location, surgical removal was indicated.

**Figure 4:**
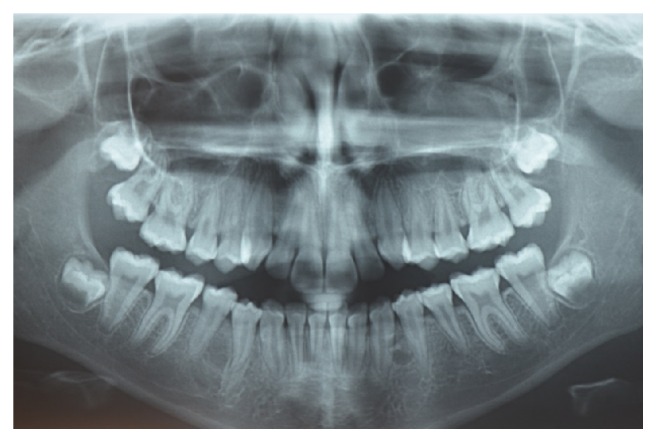
Panoramic radiograph of the patient aged 11 years and 3 months showing the early formation of a supplemental supernumerary tooth in the left mandibular premolar region.

Before beginning the second phase of the treatment, additional tests and a new panoramic radiograph were requested, which revealed the formation of a supplemental supernumerary tooth, this time in the left mandibular premolar region (Figure 4), which was also surgically removed.

**Figure 5:**
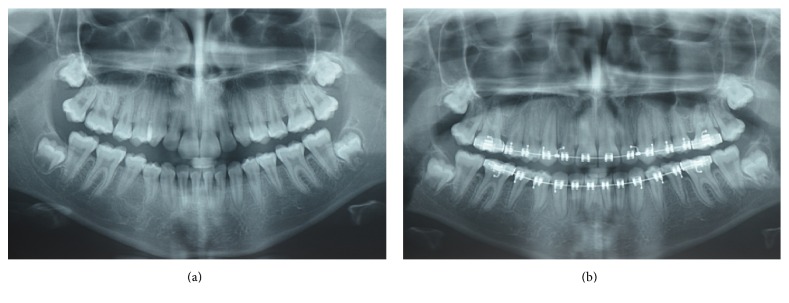
Panoramic radiographs of the patient aged (a) 12 years and 5 months and (b) 13 years and 7 months with no evidence of further supernumerary teeth.

Control panoramic radiographs taken at two different moments along the orthodontic treatment demonstrated no evidence of further supernumerary teeth (Figure 5).

**Figure 6:**
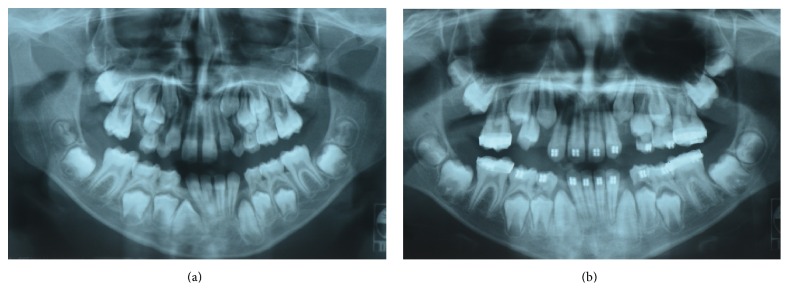
Panoramic radiographs of the patient aged (a) 9 years and 11 months and (b) 10 years and 4 months with no evidence of supernumerary teeth.

**Figure 7:**
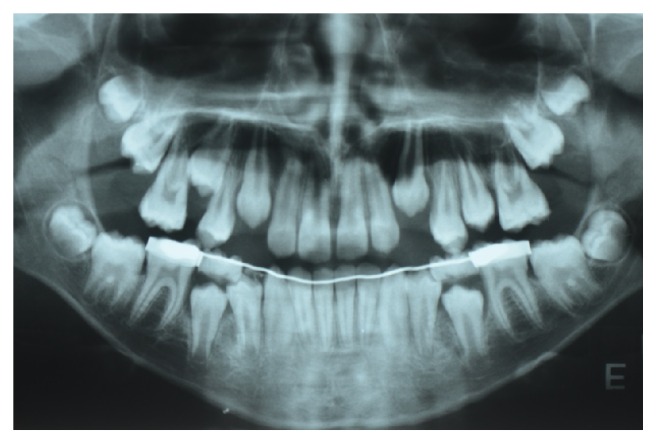
Panoramic radiograph of the patient aged 11 years and 10 months with no evidence of supernumerary teeth.

*Case  2.* A male, leucoderma, Brazilian patient, aged 9 years and 11 months, presenting Class 3 malocclusion, sought a private clinic for orthodontic treatment, which was planned in two phases (interceptive and corrective), to be completed with orthognathic surgery. Similarly to Case  1, the initial panoramic radiographs revealed no abnormalities in the dentition (Figures 6 and 7).

In the first phase of the orthodontic treatment, a modified HAAS expander associated with a facial mask was used for 8 months. Two years later, a new expander and facial mask were recommended for further 6 months.

**Figure 8:**
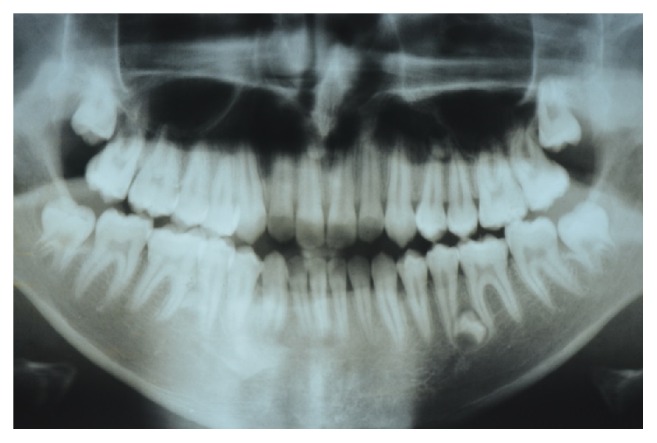
Panoramic radiograph of the patient aged 16 years showing the early formation of 3 supernumerary teeth in the right and left mandibular premolar region and in the left maxillary premolar region.

**Figure 9:**
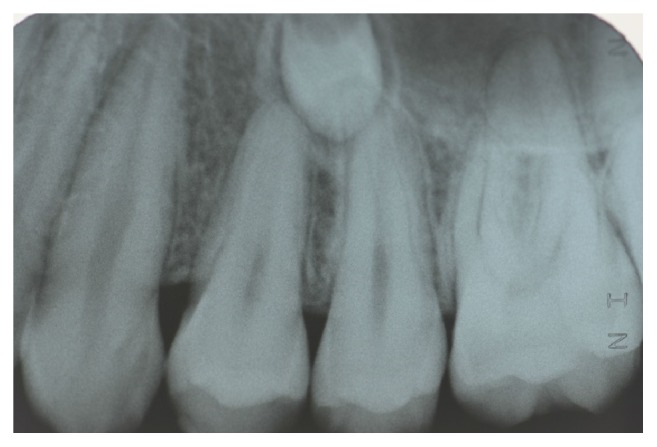
Periapical radiograph of the patient showing a supernumerary tooth in the left maxillary region.

**Figure 10:**
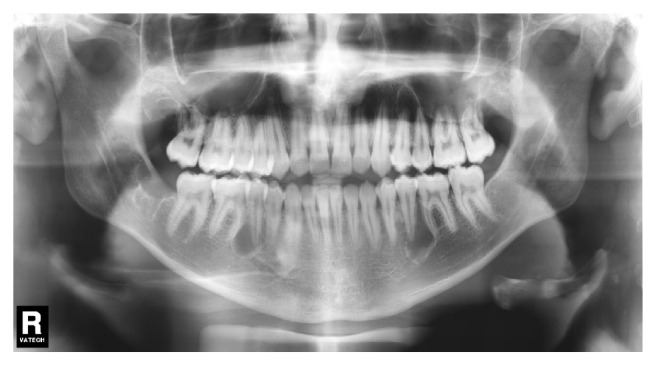
Panoramic radiograph of the patient aged 18 years after the extraction of all supernumerary teeth and third molars.

During the planning of the second phase of the treatment, with complete permanent dentition, an orthodontic/surgical approach was deemed necessary after facial growth had been completed. When additional tests were performed, the radiographic follow-up revealed the presence of three supernumerary teeth in the premolar region (Figures 8 and 9). Surgical removal of all third molars as well as the 3 supernumerary teeth was performed in a hospital setting under general anesthesia, along with the surgery for the correction of deviated nasal septum (Figure 10).

**Figure 11:**
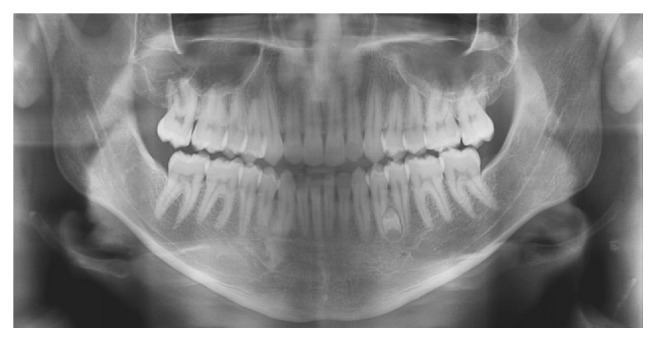
Panoramic radiograph of the patient aged 20 years and 5 months showing a supplemental supernumerary tooth in the left mandibular premolar region.

At 20 years and 5 months, during orthognathic surgery planning, a new radiographic image showed a supplemental supernumerary tooth in the left mandibular premolar region (Figure 11), which was also surgically removed.

## 3. Discussion

Supernumerary teeth may cause several disorders, such as the displacement, rotation or impaction of permanent teeth, crowding, abnormal or premature diastema, space closure, abnormal or delayed root development, and root resorption, and also lead to the formation of follicular primordial cysts [2, 5, 9, 10, 12, 13].

Currently, the most accepted theory regarding the etiology of supernumerary teeth is localized hyperactivity of the dental lamina [1, 4, 5, 7, 14]. The mobility of the facial process during facial growth can result in the rupture of the dental lamina. If these structures penetrate a region that allows their development, an enamel organ may be formed, resulting in the emergence of a supernumerary tooth. Other theories include genetic factors [15], gender and racial inheritance [5, 9, 10, 12, 13], and the combination of genetic and environmental factors [16]. More than 20 syndromes and developmental conditions appear to be associated with single and multiple supernumerary teeth such as Gardner's syndrome or Cleidocranial Dysplasia [2, 17]. However, the occurrence of supernumerary teeth associated with systemic conditions or syndromes is a rare event [11].

The most common type of supernumerary teeth to sequentially develop is the mandibular premolars [9]. Although supernumerary premolars have been reported bilaterally in both the mandible and maxilla, they occur almost three times more in the mandible than in the maxilla [5]. Supernumerary mandibular teeth tend to maintain their normal shape and size. However, in the maxilla, they are more likely to present alterations, with the conical shape being the most prevalent. Although supernumerary premolars may emerge buccally, they are usually located lingually or centrally in the alveolar ridge, which explain the reason why they tend to stay trapped [1].

Third premolars usually develop until the age of 13-14 years [1], that is, 7–11 years after the development of the normal premolars [4]. However, the root formation of third premolars has been reported to occur until the age of 23 years [1]. Although two cases of sequential supernumerary teeth have been reported in the first decade of life [9], the first sequential supernumerary premolars were identified only in the second decade [10].

When a supernumerary tooth is diagnosed, a decision must be made concerning its removal or monitoring. The possible risks and benefits of preserving or surgically extracting a supernumerary tooth must be carefully assessed in each case. Extraction of supernumerary premolars is justified due to the possibility of root resorption of adjacent teeth and the induction of pathological changes, such as cysts [3]. However, in cases when spontaneous eruption is likely, extraction should be delayed to facilitate the surgical procedure and reduce risks [1].

The recurrence of supernumerary teeth may be explained by the incomplete resorption of the dental lamina and the reactivation of a dental follicle portion at the moment when the crown of the permanent tooth is being formed [5]. The late development of supplemental teeth may also be due to the presence of supernumerary teeth crypts that were not detected in the initial radiographs [9].

In a literature review by Solares & Romero [5], the authors reported that recurrence of supernumerary premolars after their surgical removal was 8% and that patients with a previous history of supernumerary teeth in the anterior region were 24% more likely to develop supplemental premolars later. The authors also reported that 75% of all supernumerary premolars remain impacted and asymptomatic, with a 5 : 1 unerupted/erupted ratio [5]. As a result, early diagnosis of supernumerary premolar development is unlikely without timely radiographic assessment [5, 10]. Thus, clinical and radiographic monitoring of patients with previous experience of supernumerary teeth is essential for early diagnosis of supplemental premolars.

It has been previously proposed that radiographic monitoring of patients who develop supernumerary teeth should be periodically performed between 3 and 5 years. However, due to the possible complications mentioned above, it is suggested that this period is reduced to 6 to 12 months, especially in cases where the decision to keep supernumerary teeth in situ is made [5, 10].

## 4. Conclusion

Considering the late developing of supernumerary premolars, appropriate follow-up with panoramic radiographs of patients with previous experience of supernumerary teeth is essential for early diagnosis of supplemental premolars.
